# Evaluation of the Influence of Raw Almonds on Appetite Control: Satiation, Satiety, Hedonics and Consumer Perceptions

**DOI:** 10.3390/nu11092030

**Published:** 2019-08-30

**Authors:** Sophie Hollingworth, Michelle Dalton, John E. Blundell, Graham Finlayson

**Affiliations:** 1 Appetite Control and Energy Balance Research, School of Psychology, University of Leeds, Leeds LS2 9JT, UK; 2 School of Social and Health Sciences, Leeds Trinity University, LS18 5HD, Leeds LS18 5HD, UK

**Keywords:** almonds, snack, appetite, satiety, energy intake

## Abstract

Snack foods can be substantial contributors to daily energy intake, with different types of snacks exerting potentially different effects on satiety per calorie consumed. The present research compared the effect of consuming almonds as a mid-morning snack compared to an energy and weight-matched comparator snack (savoury crackers) or the equivalent weight of water (zero energy control). In a crossover design, 42 female participants (age: 26.0 ± 7.9, BMI: 22.0 ± 2.0) consumed a fixed breakfast then a mid-morning snack. Appetite, 24-h energy intake, food hedonics, and consumer perceptions of the snack foods were assessed under laboratory conditions. AUC analyses revealed a lower overall hunger drive after consuming almonds compared to crackers or water. There was no difference in 24-h energy intake in the almond compared to the cracker or the zero-energy control condition, however participants consumed more energy in the cracker condition compared to the zero-energy control condition. In addition, almonds suppressed hedonic preference (implicit wanting) for consuming high-fat foods and demonstrated a higher satiety quotient (SQ) than crackers. Almonds were perceived to have a more favourable consumer profile aligned with successful weight management. In conclusion, these findings demonstrate that in the context of a 24-h period of objectively measured energy intake, raw almonds are effective for controlling appetite compared to an energy matched alternative snack. This trial was registered at clinicaltrials.gov [NCT02480582].

## 1. Introduction

In recent years there has been a significant increase in snacking behaviour [1] with snack foods now contributing considerably more to total daily energy intake than 30 years ago [2]. This increase in snacking has occurred alongside the rise in obesity. However, it has been suggested that the relationship between increased snacking and obesity may be attributed to the types of foods typically consumed as a snack [3]. Snack foods have been characterised as having poor nutritional quality, with most consisting primarily of fats and carbohydrates [4]. If additional energy consumed from snacks is not appropriately compensated for then frequent snacking can contribute to excess energy intake [5]. On the other hand, studies have demonstrated that frequent snacking can promote feelings of satiety throughout the day and as a result lead to less overeating and improved daily energy balance [6]. Therefore, snacking is not an undesirable behaviour in itself as it can increase the opportunity for the addition or substitution of healthy foods into the diet [7].

Satiety is an important psycho-biological mechanism involved in the expression of human appetite, which inhibits hunger and intake following the consumption of a food or beverage [8]. Satiety arises through the integration of numerous factors including cognitive, sensory and post ingestive signals, conceptualised in the Satiety Cascade [9]. Foods which promote satiety have received increasing attention over recent years as satiating foods can help consumers control their appetite, eat healthily and manage their weight [10]. Importantly research demonstrates that calorie for calorie, not all foods provide the same level of satiety [11], and a hierarchy of macronutrient satiating power has been established [12] with foods that are high in protein and fibre, and low in energy density being more satiating [13,14,15]. Furthermore, the structure of food [16,17] and how an individual perceives food [18] has been shown to influence satiety.

Almonds are a food product that are high in both protein and fibre as well as fat, but relatively low in available carbohydrate and lower in digestible energy than previously considered [19]. It is well established that proteins and fibres have prominent effects on appetite control [14,20] and since they act via different mechanisms their effects may be additive. The unique structural properties and macronutrient composition of almonds may be beneficial for the control of hunger, the strength of satiety and subsequent energy intake relative to other foods.

While the exact mechanisms through which almonds might act upon appetite are unknown there is some evidence that consumption of almonds can have favourable effects on appetite control. Long-term studies have revealed that daily almond consumption does not result in significant weight change [21,22,23,24]. A recent medium-term study that compared the effects of daily almond vs. cracker snacking over eight weeks found a small change in body mass (+1kg) in both snack groups at the end of the eight weeks [25]. This change in body mass was accounted for by an increase in fat-free mass rather than body fat. Further to this, almond snacking was associated with greater glucose tolerance and whole-body insulin sensitivity [25]. In addition, acute studies have demonstrated that the addition of almonds to a meal decreases blood glucose concentrations and increases satiety in healthy adults [26,27] and in those with impaired glucose tolerance [28]. Almonds as a snack have been found to reduce both self-reported hunger and desire to eat [24]. In a recent study, a mid-morning snack of almonds (28 g and 42 g) was tested against a negative control of no almonds [29]. The authors found a portion dependent effect of almonds on subjective reports of appetite and subsequent ad libitum energy intake and overall good compensation for the calories from almonds. Consequently, the authors concluded that almonds can be a healthy snack option. To date, no acute studies have objectively assessed whether snacking on almonds leads to changes in subjective reports of appetite, subsequent objectively assessed energy intake or food hedonics (liking and wanting for food) when compared to a comparator snack which is matched for both energy and weight.

The aim of the present study was to assess the effect of consuming almonds as a mid-morning snack, compared to a weight-matched, zero energy control (water) and an energy and weight-matched comparator snack (savoury crackers) on measures of total day appetite control including appetite sensations, energy intake, food hedonics and consumer perceptions.

## 2. Materials and Methods

### 2.1. Participants

Forty-two female participants (age: 26.0 ± 7.9 years, BMI: 22.0 ± 2.0 kg/m²) were recruited from the staff and student population at the University of Leeds, UK. Participants were selected from an initial screening process to exclude those who were taking medication known to affect appetite, currently dieting to lose or maintain weight, not regular breakfast consumers, smokers, reported a history of eating disorders or were unfamiliar with or disliked the study foods. Eligible participants were invited to a screening session to confirm the inclusion criteria, have the study procedure presented to them and provide written informed consent. All procedures were reviewed and approved by the University of Leeds, School of Psychology Ethics Committee. Participants received £30 for their participation in the study.

### 2.2. Design

The study followed a randomised, counterbalanced, crossover within subjects design to examine the effects of consuming almonds compared to a weight-matched, zero energy control (water) and an energy and weight-matched comparator snack (savoury crackers) on parameters of appetite control. Participants attended the research unit on four occasions: a screening and measures session and three experimental visits. Each visit was scheduled at least seven days apart. For all visits, participants were asked to refrain from eating or drinking anything besides water from 10 pm the evening before to ensure a standardised fasted state. Compliance with this instruction was assessed at the beginning of each test session by self-report. During the experimental visits, participants were permitted to leave the research unit in the periods between meals but were instructed not to eat or drink anything besides water.

### 2.3. Measures

#### 2.3.1. Resting Metabolic Rate, Anthropometrics and Body Composition

Participants’ resting metabolic rate (RMR) was measured following an overnight fast using an indirect calorimeter fitted with a ventilated hood (GEM, Nutren Technology Ltd.). During the measure, participants were required to remain awake but motionless in a supine position for 45 min. RMR was calculated from respiratory data averaged over the final 30 min. Standing height was measured to the nearest 0.5 cm using a stadiometer. Waist circumference (cm) was measured at the participants’ naval after expiration, three measures were taken, and an average was calculated. To obtain an estimate of participants’ body composition (fat mass, fat-free mass, and percentage body fat) air plethysmography (Bodpod, Concord, CA, USA) was used. Body composition was measured whilst participants were wearing non-underwired swimwear and a swim cap. All measures were conducted following an overnight fast.

#### 2.3.2. Subjective Appetite Sensations

Subjective appetite sensations (hunger, fullness, desire to eat, and prospective consumption) were assessed using 100 mm visual analogue scales (VAS) presented on a validated Electronic Appetite Rating System (EARS-II) [30].

#### 2.3.3. Fixed Energy Breakfast

The fixed energy breakfast (muesli combined with natural yoghurt, semi-skimmed milk, and honey) served with 300 g of water, was individually calibrated to provide participants with 25% of their measured RMR. The macronutrient content of the breakfast was fixed (15% PRO, 62% CHO, 22% FAT). Participants were given 15 min to consume the breakfast in its entirety.

#### 2.3.4. Mid-Morning Snack

The amount of snack served to participants was individually calibrated with each participant being provided with 0.9 g of snack item per kg of their body weight. The amount of water provided alongside the snack was adjusted so that the total weight of the snack and water consumed equalled 300 g. The comparator snack was a supermarket brand (Sainsbury’s UK) cheese-flavoured cracker that was energy and weight-matched to the almonds (see Table 1). The mid-morning snack was consumed two hours following breakfast and participants were given 15 min to consume it in its entirety.

#### 2.3.5. Ad Libitum Energy Intake

Ad libitum energy intake was assessed using both ad libitum lunch and dinner test meals. The ad libitum lunch consisted of tomato, herb risotto, and strawberry yoghurt, and the ad libitum dinner consisted of chilli con carne and rice, garlic bread, salad, and chocolate brownies. Lunch was served four hours after breakfast and dinner was served four hours after lunch. Participants were instructed to consume as much or as little as they wanted but to eat until they reached a comfortable level of fullness. When participants left the research unit following dinner, energy intake was assessed using a take-home ad libitum snack box containing items to make a sandwich, fruit, yoghurt, crisps, and chocolate. Participants were instructed to eat from the snack box if they were hungry following the test session. All food was measured to the nearest 0.1 g pre and post-consumption and energy values were determined using manufacturer labels.

#### 2.3.6. Food Reward: Explicit Liking and Implicit Wanting for Food

The Leeds Food Preference Questionnaire [31,32] was used to assess explicit liking and implicit wanting for a selection of food images either high (>40% energy) or low (<20% energy) in fat. The mean for low-fat scores was subtracted from the mean for high-fat scores to produce an Appeal Bias for high versus low-fat food.

#### 2.3.7. Palatability Ratings and Consumer Perceptions of the Snack Foods

Participants were asked to rate the snack foods according to ‘How strong is your desire to eat more?’ ‘How difficult was it to consume the snack?’ ‘How suitable was the portion size for a snack?’ and ‘How much more could you eat of the snack?’ using 9-point Likert scales, immediately post-consumption. Participants’ perceptions of the snack items were rated of completion on the study prior to debriefing using 7-point Likert scales. Participants were asked to rate the snack items according to their pleasantness, taste, healthiness, fat and energy content, satiating capacity, association with successful weight management and desire to overconsume.

#### 2.3.8. Satiety Quotient

The satiety quotient (SQ) is a measure of the satiating capacity of foods relative to energy content. The SQ has been validated in previous research [33,34,35]. Hunger VAS ratings were used to calculate SQ for the different mid-morning snacks to assess their satiating efficiency. A higher SQ represents a greater satiating capacity per kilocalorie of an ingested food, whereas a lower SQ represents a weaker satiating capacity. The following formula was used to calculate satiety quotient:

$$\mathrm{SQ}\;\left(\frac{\mathrm{mm}}{\mathrm{kcal}}\right)=\left(\frac{\mathrm{rating}\;\mathrm{before}\;\mathrm{eating}\;\mathrm{episode}-\mathrm{rating}\;\mathrm{after}\;\mathrm{eating}\;\mathrm{episode}}{\mathrm{Energy}\;\mathrm{content}\;\mathrm{of}\;\mathrm{the}\;\mathrm{food}\;\mathrm{consumed}}\right)\times 100$$

### 2.4. Procedure

**Figure 1:**
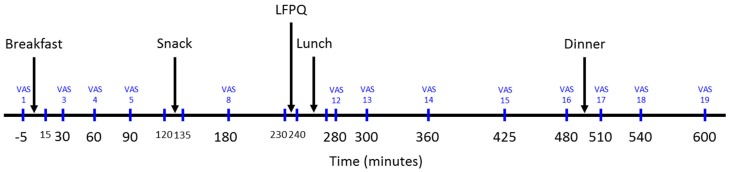
Schematic representation of the study procedure–experimental session. VAS–Visual Analogue Scale. LFPQ–Leeds Food Preference Questionnaire.

Participants arrived at the research unit between 8:00–9:00 am following an overnight fast for all sessions. In the screening and measures session, participants were provided with an explanation of the research, participants were told the purpose of the study was to ‘Examine the effect of consuming a mid-morning snack on mood throughout the day’. Participants were given the opportunity to ask any questions before providing written informed consent. Measures of resting metabolic rate, anthropometrics, and body composition were then completed. In the experimental sessions, participants completed baseline appetite ratings before consuming the fixed energy breakfast. Following breakfast, participants were free to leave the research unit but were asked to return two hours later for the mid-morning snack. Following the snack, participants left the research unit and returned two hours later for lunch. Participants completed a food preference assessment before the lunch test meal. Participants received an ad-libitum dinner 4-h following lunch and took an ad libitum snack box home at the end of the test session. Appetite ratings were completed at 30-min intervals until the mid-morning snack and then every 60-min, as well as before and after each event in the procedure. See Figure 1 for a schematic representation of the experimental session procedure.

### 2.5. Data Analyses

Data were analysed using Statistical Programme for Social Sciences (SPSS) Version 22 (SPSS Inc., Chicago, IL, USA.) and are presented as means with standard deviations. The effect of mid-morning snack condition on each appetite sensation was assessed using 3 × 19 repeated measures ANOVAs (see Table S1). To examine the effect of mid-morning snack condition on AUC appetite variables (trapezoid method), energy intake and food reward one-way repeated measures ANOVAs were conducted. The SQ for the mid-morning snacks was assessed over time using a two-way repeated-measures ANOVA. Both the Atwater (595 kcal/100 g [36]) and digestible (460 kcal/100 g [19]) energy values were used in the calculation of SQ for almonds. In addition, paired sample t-tests were used to assess palatability and perception ratings of the mid-morning snacks. Where appropriate Greenhouse-Geisser probability levels were used to adjust for non-sphericity. Where significant effects were obtained post hoc analyses, with a Bonferroni correction for multiple comparisons were conducted. An *α*-level of 0.05 was used to determine significance and Cohen’s d was used as a measure of effect size.

## 3. Results

### 3.1. Overall Sample Characteristics

Characteristics of age, anthropometric measures, body composition and psychometric traits for the overall sample are shown in Table 2. Of the 42 participants who completed the study, 10% were overweight, 69% were Caucasian, and 26% were not University students.

### 3.2. Subjective Appetite Sensations

Baseline ratings of hunger (*p* = 0.94), fullness (*p* = 0.83), desire to eat (*p* = 0.45) and prospective consumption (*p* = 0.73) did not differ significantly across the three snack conditions. There was an interaction between snack condition and time on ratings of hunger (F(36,1476) = 9.81, *p* < 0.001), desire to eat (F(36,1476) = 7.35, *p* < 0.001), prospective consumption (F(36,1476) = 7.65, *p* < 0.001) and fullness (F(36,1476) = 7.55, *p* < 0.001). Post-hoc analyses revealed that ratings of hunger and desire to eat were lower immediately following the snack in the almond compared to the cracker (*p* = 0.005, *p* = 0.049) and water condition (both *p* < 0.001) and in the cracker compared to the water condition (both *p* < 0.001). Ratings of hunger, desire to eat and prospective consumption were higher, and ratings of fullness were lower in the water condition compared to the almond and cracker condition in the 2-h period before lunch (all *p* < 0.001). Ratings of desire to eat were higher in the cracker condition compared to the almond condition 2-h after lunch (*p* = 0.019).

**Figure 2:**
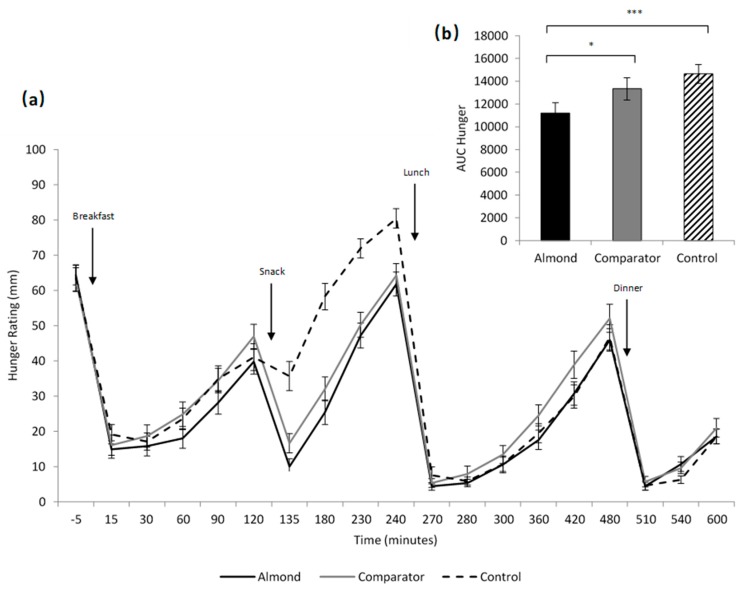
Subjective ratings of hunger (**a**) and area under the curve hunger (**b**) for the three different snack conditions. * *p* < 0.05; *** *p* < 0.001.

There was effect of snack condition on AUC hunger (F(2,82) = 14.72, *p* < 0.001), AUC fullness (F(2,82) = 5.93, *p* < 0.01), AUC desire to eat and AUC prospective consumption (F(2,82) = 10.43, *p* < 0.001) across the test day. AUC hunger (see Figure 2), AUC desire to eat, and AUC prospective consumption were lower in the almond condition compared to the water (*p* < 0.001, *d* = 0.6, *p* < 0.001, *d* =0.6, *p* < 0.001, *d* = 0.5) and cracker condition (*p* = 0.012, *d* = 0.4, *p* = 0.019, *d* = 0.3, *p* = 0.042, *d* = 0.3). There was no difference in AUC hunger, AUC desire to eat, or AUC prospective consumption in the cracker compared to the water condition (*p* = 0.46, *p* = 0.14, *p* = 0.29, respectively). AUC fullness was higher in the almond compared to the water condition (*p* = 0.003, *d* = 0.3) and there was no difference in the cracker condition compared to the almond (*p* = 0.07) or water condition (*p* = 1).

### 3.3. Ad Libitum Energy Intake

**Figure 3:**
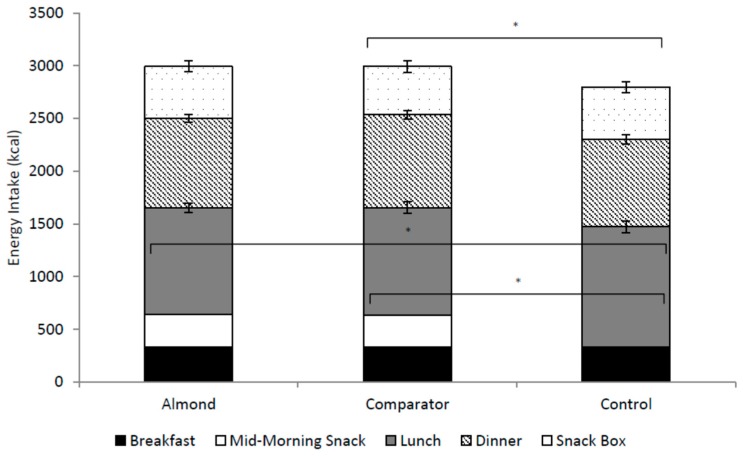
Total energy intake (kcal) for the different snack conditions. * *p* < 0.05.

Figure 3 shows participants’ overall energy intake across the test day, consisting of breakfast, snack, lunch, dinner, and the snack box. There was a main effect of snack condition on ad libitum energy intake at lunch (F(2,82) = 7.04, *p* < 0.001). Participants consumed fewer calories during the lunch test meal in the almond (M: 1007.3, SD: 299.1, *p* = 0.004, *d* = 0.4) and the cracker (M: 1019.6, SD: 345.6, *p* = 0.014, *d* = 0.4) conditions compared to the water condition (M: 1143.6, SD: 347.4). There was no difference in calorie intake during the lunch test meal in the almond compared to the cracker condition (*p* = 0.76, *d* = 0.04). There was a main effect of snack condition on total energy intake (F(2,82) = 4.29, *p* < 0.05). Post hoc analyses revealed that participants consumed a greater total amount of calories in the cracker condition (M: 2990.6, SD: 748.9) compared to the water condition (M: 2797.2, SD: 728.2, *p* < 0.05, *d* = 0.3). However, total energy intake did not differ significantly in the almond condition (M: 2992.0, SD: 654.2) compared to the water condition. Energy intake at the ad libitum dinner (F(2,82) = 1.66, *p* >0.05) and from the snack box (F(2,82) = 0.46, *p* > 0.05) did not differ by snack condition.

### 3.4. Food Reward-Explicit Liking and Implicit Wanting: Fat Appeal Bias

For explicit liking there was a main effect of snack condition (F(2,82) = 3.26, *p* < 0.05) with a lower appeal bias for high fat foods in the cracker condition compared to the water condition (*p* < 0.05, *d* = 0.3). Furthermore, for implicit wanting there was a main effect of snack condition (F(2,82) = 4.67, *p* < 0.05) with a lower appeal bias for high fat foods in the almond condition (*p* < 0.02, *d* = 0.3) and the cracker condition (*p* < 0.05, *d* = 0.4) compared to the water condition (see Table 3).

### 3.5. Satiety Quotient

**Figure 4:**
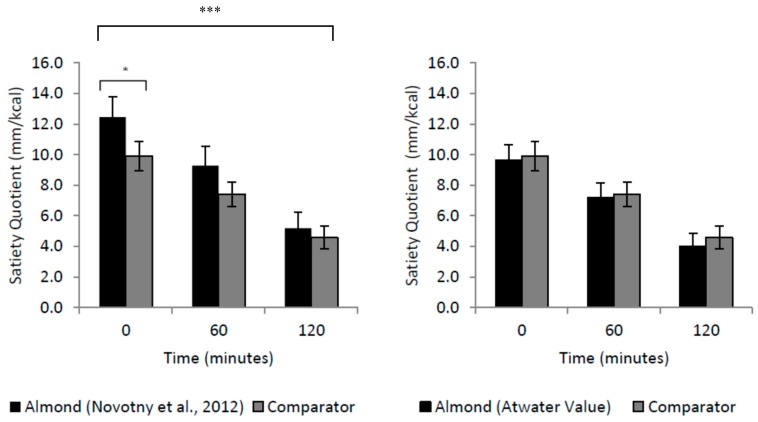
Satiating efficiency of the snacks, 120 min post consumption. * *p* < 0.05; *** *p* < 0.001.

Figure 4 shows the satiating efficiency of the mid-morning snacks using both the Atwater and the digestible energy [19] values to calculate the satiety quotient for almonds. When using the digestible energy value of almonds there was no effect of snack condition (F(1,41) = 2.69, *p* = 0.109). There was a main effect of time (F(1,54) = 101, *p* < 0.001), the satiating efficiency of both mid-morning snacks decreased over the 120-min period post consumption. In addition, there was an interaction between snack condition and time (F(1,58) = 4.27, *p* < 0.05). Post hoc comparisons revealed that when using the digestible energy value of almonds to calculate the satiety quotient, satiating efficiency immediately post consumption, was greater for almonds compared to that of the crackers (*p* < 0.05, *d* = 0.3). When using the Atwater values, there was no effect of snack condition (F(1,41) = 0.183, *p* = 0.67). There was a main effect of time (F(2,82) = 97.0, *p* < 0.001) with the satiating efficiency of both mid-morning snacks decreasing over the 120-min post consumption. There was no interaction between snack condition and time (F(2,82) = 0.199, *p* = 0.82).

### 3.6. Palatability and Perceptions of the Mid-Morning Snack

See Table 4 for the palatability and perception ratings of the almond and comparator snack. Immediately following consumption of the mid-morning snack desire to eat more of that snack was lower in the almond compared to the cracker condition (*p* < 0.01, *d* = 0.5). Participants rated the almond snack as more difficult to chew (*p* < 0.001, *d* = 1.1) and rated the portion size as too large (*p* < 0.01, *d* = 0.6) compared to the crackers. Additionally, when asked how much more they could eat of the snack, the response was lower for the almonds compared to the cracker condition (*p* < 0.05, *d* = 0.4). Following the completion of the study, the mid-morning snacks were rated as equally palatable and habitual consumption of the different snack items did not differ. Almonds were perceived as healthier (*p* < 0.001, *d* = 3.1), lower in fat (*p* < 0.05, *d* = 0.4), and calories (*p* < 0.05, *d* = 0.4) and more filling (*p* < 0.001, *d* = 0.9) compared to crackers. In addition, almonds were rated higher with regards to aiding successful weight management (*p* < 0.001, *d* = 2.2) and lower for the likelihood of overconsumption (*p* < 0.001, *d* = 1.0) compared to crackers.

## 4. Discussion

The present study aimed to examine the appetitive effects of almonds consumed as a mid-morning snack in comparison to an energy matched comparator snack. To do this the study assessed the acute satiating effects of a mid-morning almond snack compared to a weight-matched, zero-energy control (water), and an energy and weight-matched comparator snack (savoury crackers) in healthy females. This was the first study conducted to assess the effect of consuming an almond snack compared to a comparator, matched for both energy and weight under controlled laboratory conditions. The present study also considered the effects of consuming almonds on liking and wanting for subsequent high- or low-fat foods and consumer perceptions. Results indicated that in the context of a 24-h period of objectively measured energy intake, almonds were effective for controlling appetite and lowering the hedonic preference for high-fat foods to at least the same degree as an energy matched comparator.

As expected, both the almond and the cracker snack suppressed hunger more than the water. However, compared to the comparator snack, participants exhibited lower hunger and desire to eat after consuming the almonds. This effect may be attributable to the high protein and fibre content of the almonds, both of which are highly satiating nutrients associated with the release of appetite-related hormones [13,14]. Furthermore, the effect may have been mediated by differences in the ease of consumption (e.g., oral mechanical effort) between almonds and crackers. Greater chewing of almonds has been shown to result in greater suppression of hunger in conjunction with increased concentrations of post-prandial glucagon-like peptide-1 and reduced decline in insulin [37]. Future studies should assess biomarkers of satiety alongside behavioural measures to help establish the mechanisms by which almonds affect eating behaviour and appetite control. In addition to the reduced hunger, almonds had a higher satiety quotient than the crackers. When satiety quotient was calculated using the Novotny et al. (2012) energy value for almonds, the almonds generated a greater suppression of hunger per calorie consumed. However, it is important to note there was no difference compared to the crackers when using the Atwater energy value for almonds. One possible explanation for the almonds greater satiating potential is the gradual release of lipids into the small intestine, which causes GLP-1 secretion and as a result a reduced appetite [38]. Future research could consider blood lipid levels to investigate this further.

Following consumption of the almond and comparator snack, we found that participants partially compensated by adjusting their energy intake at the ad libitum lunch. However, energy intake from the ad libitum dinner and the ad libitum snack box did not differ significantly from the zero-energy control. This finding is only partly consistent with previous research [29] which demonstrated that participants adjusted their food intake in response to an almond snack at both lunch and dinner (albeit to a lesser extent at dinner). However, taken together these findings demonstrate that consuming almonds as a mid-morning snack has acute beneficial effects on appetite control. The lower energy intake at lunch following the mid-morning snacks demonstrated in the present study was consistent with participants’ self-reported appetite sensations, as participants exhibited greater levels of fullness, and lower levels of hunger, desire to eat, and prospective consumption in the post snack satiety phase. This complements previous research that has demonstrated appetite sensations to be associated with subsequent energy intake [34]. When overall test day energy intake was examined, there were no differences in energy consumed in the almond snack condition compared to the comparator snack condition. We did find that participants consumed more energy on the comparator snack day compared to the zero-energy control, however, the effect size was small. Conversely, overall test day energy intake did not differ significantly in the almond condition compared to the zero-energy control condition, suggesting that almonds can be incorporated into the diet without the addition of surplus energy. What is more, it has been proposed that a significant proportion of the nutrients contained in almonds are not bio-accessible [19,23,39], therefore the actual energy absorbed from the almonds in the current study may be lower than the reported total energy consumed (which were calculated using the Atwater energy values rather than the adjusted energy values based on [19]). Further research on the bio-availability of energy from almonds (and other foods) is required to support this suggestion, however, these findings are consistent with longer-term studies of almond consumption that demonstrate regular almond intake was not associated with weight gain [21,22,23,24].

When participants’ hedonic preference for consuming high-fat versus low-fat foods was examined, we found that the mid-morning snack of almonds significantly reduced the degree to which participants wanted high-fat foods compared to the control condition. This finding is of importance as previous research has shown that increased wanting for high-fat food is associated with greater binge eating tendencies and greater overall energy intake [40]. Furthermore, it is the first to suggest that almonds have beneficial effects on hedonic risk factors for overconsumption (i.e., reducing wanting for high-fat foods).

The final aim of the current study was to explore participants’ perceptions of almonds compared to crackers when consumed as a snack food. Our findings suggest that participants’ perceived almonds as having a greater satiating potential with almonds being perceived as healthier, lower in fat and calories, more filling, less associated with overeating, and more favourable for weight management compared to crackers. Research has demonstrated that expectations about the satiating effects of food play a role in satiety [41,42]. Participants also perceived almonds as more difficult to chew. This did not appear to reflect the pleasantness of the snack as the almonds and comparator snack were rated as equally palatable, rather than due to the texture of the almonds. The texture and chewiness of almonds may represent an additional mechanism behind their greater satiating capacity, with evidence suggesting that oral processing plays an important role in food intake by affecting both satiation and satiety [17,43].

Overall these results confirm the proposed action, unique to almonds, according to different mediating processes involved in the development of satiety. The mechanisms identified include cognitive (perceptions and expected satiety), sensory (oral exposure time), post-ingestive (lower hunger drive and preference for high-fat foods), and pre-absorptive factors (metabolisable energy content of almonds). While independently we would expect these actions to be relatively subtle, almonds may be particularly beneficial due to their combined action. It is proposed that repeated consumption of almonds as a snack in the diet may have long-term benefits on appetite control. Especially when considering that long-term studies indicate no reduction in the acute satiety effect of regular almond consumption [21,23,24] therefore effects on short-term satiety are likely to persist following habitual consumption. This may be especially true in individuals who find it difficult to control their hunger, for instance, individuals who experience low satiety responsiveness may benefit from incorporating almonds into their diet [35]. It is important to note that the almonds used in the current research were whole, raw almonds and the results should be confirmed for almonds that have been processed by roasting and/or flavour enhancements. Evidence suggests that the bio-accessibility of nutrients from roasted almonds is greater compared to whole raw almonds [44,45] and that roasted almonds are associated with decreased chewing, smaller fragments, and lower maximum bite force [45,46,47]. However, the addition of almonds to the diet in different forms (including whole raw almonds, roasted almonds, almond butter) has been shown to have positive effects on cholesterol [48,49] and lipid profiles [48], and are not associated with significant changes in body weight [24,25,45,48].

Despite the present study carrying several strengths, there are some limitations to consider. While participants were instructed not to engage in physical activity for 24 h prior to each experimental session, compliance was assessed using self-report and physical activity was not actually measured. Future studies should consider controlling for this by objectively measuring habitual physical activity levels, and/or physical activity around the study test days. Also, a measure of physical activity level could be included in the estimation of individual energy requirement. Additionally, while the results are representative of this study sample, which was healthy weight females, they may not be replicated in other populations. These findings should be confirmed in an overweight obese sample, as typically such individuals are poorer at identifying and responding to appetite sensations and compensating for additional energy in the diet [50,51,52]. A recent systematic review and meta-analysis showed that nut consumption (not specific to almonds) was associated with increased energy intake, but not weight gain in overweight and obese individuals [53]. However, much of the energy intake data reported in this meta-analysis relied on self-reported food diaries and only six studies were included in the analysis. Consequently, it is not known whether the ability to compensate accurately for an almond snack is compromised in overweight and obese individuals. Finally, while the comparator snack food was energy-matched to almonds other macronutrients were not matched across the comparator and almond snack. The difference in macronutrient profiles was intentional in the design and is not a flaw of the current study however it should be noted that previous research has shown that when consumption of whole, lightly roasted salted almonds were compared to an energy- and macronutrient-matched baked food there was no differential effect on postprandial appetite or neural responses to food images [54]. Taken together, the findings of the current study and of Sayer et al. [54] supports the suggestion that macronutrients, and by extension, the macronutrient profile of almonds is important to their appetitive effects.

## 5. Conclusions

This study has shown that the addition of almonds to the diet as a mid-morning snack reduced overall hunger and subsequent ad libitum energy intake and reduced hedonic wanting for high-fat foods when compared to a weight-matched, zero-energy control and an energy and weight-matched comparator snack. These findings suggest that almonds are an appropriate snack food to incorporate into the diet. With the increasing prevalence of overweight and obesity, dietary strategies such as the inclusion of snacks that promote satiety are particularly practicable.

## Figures and Tables

**Table 1:** Nutritional composition of the almond (based on Atwater values) and comparator snack.

Item	KCAL/100 g	FAT/100 g (%)	CHO/100 g (%)	PRO/100 g (%)
Almonds	595	49.9 (75.5)	9.1 (5.7)	21.2 (14.2)
Savoury Crackers	581	40.6 (62.9)	38.3 (24.7)	14.2 (9.8)

**Table 2:** Mean (SD) for age, anthropometrics, body composition and trait characteristics.

	Mean (Standard Deviation)
Age (years)	26.0 (7.9)
Weight (kg)	58.5 (6.1)
BMI (kg/m^2^)	22 (2.0)
Waist (cm)	73.5 (5.6)
Fat Mass (kg)	15.4 (5.0)
Fat Free Mass (kg)	43.0 (4.1)
Body Fat (%)	26.0 (6.7)
TFEQ Restraint	9.4 (4.8)
TFEQ Disinhibition	7.6 (2.8)
TFEQ Hunger	6.1 (3.2)

**Table 3:** Mean (SD) explicit liking and implicit wanting fat appeal bias for the different snack conditions.

	Mean (Standard Deviation)
Explicit liking	
Almond	7.8 (17.2)
Comparator	6.6 (18.3)
Control	11.7 (17.3)
Implicit wanting	
Almond	10.6 (30.6)
Comparator	5.5 (40.1)
Control	20.3 (27.7)

**Table 4:** Mean (SD) palatability and perception ratings for the almond and comparator snack.

	Almond	Comparator	*p* value
How strong is your desire to eat more?	2.4 (2.2)	3.6 (2.4)	*p* < 0.01
How difficult was it to consume the snack?	5.1 (2.6)	2.7 (1.9)	*p* < 0.001
How suitable was the portion size?	7.4 (1.4)	6.6 (1.5)	*p* < 0.01
How much more could you eat of the snack?	2.2 (1.6)	2.8 (1.7)	*p* < 0.05
How often do you consume this kind of snack?	3.1 (1.4)	2.8 (1.1)	*p* = 0.24
How pleasant was the taste of the snack?	4.7 (1.7)	5.2 (1.5)	*p* = 0.12
How sweet was the snack?	2.8 (1.5)	1.8 (1.2)	*p* < 0.001
How intense was the snack?	3.8 (1.5)	4.5 (1.4)	*p* < 0.05
To what extent do you think the snack is healthy?	6.0 (1.0)	2.4 (1.3)	*p* < 0.001
To what extent do you think the snack is high fat?	4.3 (2.1)	5.1 (1.5)	*p* < 0.05
To what extent do you think the snack is high calorie?	4.5 (2.1)	5.2 (1.5)	*p* < 0.05
How filling do you consider the snack to be?	5.4 (1.2)	4.2 (1.4)	*p* < 0.001
To what extent do you associate this snack with successful weight management?	5.2 (1.6)	2.1 (1.3)	*p* < 0.001
To what extent do you associate this snack with consuming too much?	3.1 (1.9)	4.9 (1.7)	*p* < 0.001

## Supplementary Materials

The following are available online at https://www.mdpi.com/2072-6643/11/9/2030/s1, Table S1: F-ratio and *P*-values for the 3 × 19 repeated measures ANOVAs for subjective appetite ratings.
