# Supplementary material for: Evaluation of the Influence of Raw Almonds on Appetite Control: Satiation, Satiety, Hedonics and Consumer Perceptions
Source: Nutrients. 2019 Aug 30;11(9):2030. doi: 10.3390/nu11092030 (PMC6769453; doi:10.3390/nu11092030)
Supplement: Supplementary file 1 [file nutrients-11-02030-s001.pdf]

Supplementary Table 1: F-ratio and *P*-values for the 3×19 repeated measures ANOVAs for **subje**ctive appetite ratings.

3 × 19 repeated measures ANOVA			
	Main effect snack	Main effect time	Interaction snack x time
Hunger	$F(2,82) = 12.98, p < 0.001$	$F(18,738) = 149.57, p < 0.001$	$F(36,1476) = 9.81, p < 0.001$
Fullness	$F(2,82) = 7.72, p < 0.001$	$F(18,738) = 126.95, p < 0.001$	$F(36,1476) = 7.55, p < 0.001$
Desire to eat	$F(2,82) = 18.17, p < 0.001$	$F(18,738) = 135.98, p < 0.001$	$F(36,1476) = 7.35, p < 0.001$
Prospective consumption	$F(2,82) = 11.40, p < 0.001$	$F(18,738) = 124.11, p < 0.001$	$F(36,1476) = 7.65, p < 0.001$.
